# Outcomes of Strabismus Surgery in Patients with Cranial Nerve Palsy

**DOI:** 10.3390/jcm14207221

**Published:** 2025-10-13

**Authors:** Laetitia Hinterhuber, Sandra Rezar-Dreindl, Ursula Schmidt-Erfurth, Eva Stifter

**Affiliations:** Department of Ophthalmology and Optometry, Medical University of Vienna, Währinger Gürtel 18-20, 1090 Vienna, Austria; laetitia.hinterhuber@meduniwien.ac.at (L.H.); sandra.rezar-dreindl@meduniwien.ac.at (S.R.-D.); ursula.schmidt-erfurth@meduniwien.ac.at (U.S.-E.)

**Keywords:** strabismus surgery, cranial nerve palsy, third nerve palsy, fourth nerve palsy, sixth nerve palsy, surgical outcomes

## Abstract

Strabismus, or squint or deviating eyes, is defined as misalignment of the eyes when fixating on an object and is a common problem in ophthalmology. Palsy of the third, fourth or sixth cranial nerve is one of the leading underlying causes for paralytic strabismus, often requiring surgery. However, uncertainty regarding factors influencing surgical success remains. **Background/Objectives:** The purpose of this study is to review the outcome and influencing factors of strabismus surgery in patients with cranial nerve palsy. **Methods**: A retrospective study of 57 patients with third cranial nerve (CN3) palsy, fourth cranial nerve (CN4) palsy, sixth cranial nerve (CN6) palsy or combined nerve palsy who underwent strabismus surgery between October 2009 and December 2023 was conducted. Analyzed data included demographic details, type of surgical intervention, etiology of nerve palsy, pre- and postoperative angle of deviation (AOD), vertical deviation (VD), best-corrected visual acuity (BCVA), and refractive error. **Results**: Mean age was 41.29 ± 23.14 years with a mean follow-up of 10.8 ± 15.38 months. 30 patients (52.63%) had CN6 palsy, 12 patients (21.05%) had CN3 palsy, eight patients (14.04%) had CN4 palsy and seven patients (12.28%) had combined nerve palsy. Brain neoplasm was the most common cause of nerve palsy (33.33%). Mean preoperative AOD improved from 17.54° ± 10.68 to 7.13° ± 8.93 and from 17.21° ± 9.58 to 7.49° ± 9.75 for near and distance, respectively (*p* < 0.001). Changes in VD, refractive error, and BCVA were not statistically significant. **Conclusions**: Age, gender, preoperative AOD, subtype and etiology of nerve palsy had no significant influence on surgical outcomes, which are satisfactory in patients with cranial nerve palsy (80.7%).

## 1. Introduction

The treatment of paralytic strabismus caused by extraocular muscle palsy still poses a challenge due to the complex pathophysiology. Paralytic strabismus is defined by a lesion along the pathway of the cranial nerves III (CN3), IV (CN4), and VI (CN6), either due to congenital causes or secondary to trauma, inflammation, neoplasms, or aneurysms [1,2,3]. As multiple nationwide cohort studies have shown, the incidence rate increased with age and was higher in men than in women [4,5].

If the nerve palsy is complete, oculomotor or third nerve palsy leads to incomitant strabismus and a fixed eye position in abduction, infraduction, and intorsion with a ptotic eyelid. Partial oculomotor nerve palsy results in varying paretic states of the affected muscles [6,7,8]. CN3 palsy accounts for one-third of the cranial nerve palsies and is the most challenging to operate on due to the large number of innervated muscles [9].

The fourth cranial nerve, the trochlear nerve, supplies the superior oblique (SO) muscle. Affected patients experience vertical or torsional diplopia and hypertropia, with potential esotropia in the affected eye [10,11]. Treatment options include the recession of the inferior oblique (IO) muscle or tucking the SO tendon [12].

Abducens or sixth nerve palsy is the most common cause of paralytic strabismus in adults. It affects the lateral rectus (LR) muscle and results in horizontal diplopia and esodeviation [13]. Surgery is indicated if the esodeviation does not resolve naturally and remains stable for more than six months, since it often leads to unnatural compensatory head positions [14,15].

Strabismus in adults has multiple functional impacts, including diplopia, abnormal head postures, and difficulties in distance estimation and visual orientation [16,17]. Numerous studies have shown the adverse effects on the quality of life as strabismus is associated with decreased self-esteem, higher levels of depression, anxiety, and social phobia [16,18,19,20]. In children and adolescents, paralytic strabismus can disrupt functional vision development and school activities, leading to social discrimination and an increased vulnerability to alcohol use, anxiety, and depression [21,22].

The burden of paralytic strabismus has been further highlighted in a nationwide cohort study from France, reporting higher reoperation rates compared to non-paralytic strabismus [23].

Surgery has been described as an effective treatment for strabismus due to cranial nerve palsy, but uncertainties remain concerning the influencing factors of surgical outcomes and the ideal approach. Therefore, this study aims to assess the existing surgical procedures for treating strabismus due to oculomotor, trochlear, and abducens nerve palsy.

## 2. Materials and Methods

Medical records of patients with diagnosed nerve palsy were retrospectively reviewed at the Department of Ophthalmology and Optometry of the Medical University of Vienna. Patients who received strabismus surgery between October 2009 and December 2023 due to CN3, CN4, and CN6 palsy were included in this study, while patients with a history of strabismus caused by severe head injury were excluded. Severe head injury was defined as trauma with documented loss of consciousness >24 h or cases requiring neurosurgical management. History of systemic diseases associated with strabismus led to exclusion. These were defined as endocrine disorders (Graves’ disease), genetic syndromes (Down syndrome), neuromuscular disorders (myasthenia gravis, muscular dystrophies), and neurological syndromes (Duane syndrome, Moebius syndrome).

Patients diagnosed with strabismus due to cranial nerve palsy who did not have documented follow-up data after surgery were also excluded.

Demographic information included age at the time of surgery and gender. Collected outcome measures entailed the mean angle of deviation (AOD) in near and distance, best-corrected visual acuity (BCVA), vertical deviation (VD) in near and distance, refractive error, type of etiology and surgical approach. Preoperative data were analyzed and compared to the first follow-up after one month or less and the second follow-up after at least three months following surgery. Due to the study’s retrospective nature, a few patients had follow-up periods that slightly deviated from the general time frame of the two follow-ups.

The primary outcome was successful alignment, defined as a postoperative AOD of ≤10 degrees. AOD was evaluated in the primary position by using the alternative prism cover, which was set at a distance of 33 cm (near) or 6 m (distance), or by implementing the modified Krimsky’s test if patients could not cooperate due to poor vision. Values noted in prism diopters (PD) were transformed into degrees using Lachenmayr et al.’s [24] method, which converts 1.75 PD into 1°. All subjects were assessed in a complete ophthalmological examination at each visit. Visual acuity (VA) was examined after cycloplegic refraction. The refractive error was corrected before surgery.

The severity of cranial nerve palsy was based on the documentation in the medical records and classified as either complete or partial, as noted by the treating ophthalmologist. Due to the retrospective nature of this study, no additional grading system was applied.

The study adheres to the tenets of the Declaration of Helsinki and obtained approval from the Ethics Board of the Medical University of Vienna (EK Nr. 1260/2024). In accordance with the Ethics Board of the Medical University of Vienna, informed consent was not required from the participants due to the pseudonymization of patient data and the retrospective study design.

The statistical analysis was conducted using Microsoft Excel and SPSS (IBM Statistics, Version 29). *p*-values ≤ 0.05 were considered statistically significant. For outcomes that did not reach statistical significance, effect sizes were reported. For parametric tests, Cohen’s d with corresponding 95% confidence interval was stated, for non-parametric tests, effect size r was documented. The format mean ± standard deviation (SD) or median and interquartile range (IQR) was used to describe continuous variables. Categorical variables are shown as counts and percentages. BCVA was calculated as the logarithm of the minimal angle of resolution (logMAR). Pre- and postoperative data were evaluated using Student’s *t*-test or Wilcoxon test, while influencing factors of the surgical outcome were analyzed through multivariable logistic regression.

## 3. Results

### 3.1. Baseline Characteristics

Mean age of the included 57 patients was 41.29 ± 23.14 years, ranging from 1.27 to 75.43 years. The male-to-female ratio was 30:27. The left eye was affected in 25 (43.86%) cases, the right eye in 27 (47.37%) cases and five patients had bilateral nerve palsy (8.77%). Mean total follow-up was 10.8 ± 15.38 months.

Seven (12.28%) patients presented with combined nerve palsy, whereas the remaining 50 (87.77%) patients had isolated nerve palsy. Out of the seven patients with combined nerve palsy, four (57.14%) subjects had a combination of CN6 and CN3 palsy, two (28.57%) subjects had a combination of CN3 and CN4 palsy and one (14.29%) had combined CN4 and CN6 palsy. Out of the 50 isolated cases, CN6 palsy was the most common nerve palsy with 30 (60%) cases, followed by CN3 palsy with 12 (24%) and CN4 palsy with eight (16%) patients.

Etiologies were classified as congenital in two (3.51%), acquired in 47 (82.46%) and unknown in eight (14.04%) cases. The causes of the acquired nerve palsies were further subdivided into inflammation in one (2.13%), trauma in 13 (27.66%), brain neoplasm in 19 (40.43%) and vascular disease in 14 (29.79%) patients. The etiologies classified according to the type of nerve palsy are summarized in Table 1.

Surgery was carried out under general anesthesia with a limbal approach and without any adjustable sutures. In patients with CN3 palsy, two-muscle surgery included LR recession and medial rectus (MR) plication. In patients with CN6 palsy, MR recession and LR plication were predominantly done. In cases of hypertropia due to CN4 palsy, weakening procedures for the inferior oblique (IO) muscle were most commonly performed. The method and surgical dosage were chosen individually according to the preoperative AOD. The different procedures according to the type of nerve palsy are listed in Table 2. Table 3 summarizes the applied surgical dosages.

Postoperative outcome was considered successful with a residual AOD ≤ 10° and was obtained in 46 subjects (80.7%). Subjects with isolated CN6 palsy had the best success rate (90%), followed by participants with isolated CN4 palsy (87.5%), combined nerve palsy (71.43%) and lastly isolated CN3 palsy (58.33%). Table 4 provides an overview of the patients’ demographic factors and clinical characteristics.

### 3.2. Change in Angle of Deviation

Mean preoperative AOD measured in near in patients with isolated CN3 palsy improved from 23.48° ± 14.7 preoperatively to 11.5° ± 12.8 at first follow-up and to 13.44° ± 15.61 at second follow-up. AOD measured in distance changed from 20.93° ± 14.24 to 10.57° ± 14.72 at the first follow-up and to 12.14° ± 17.15 at the second follow-up. Both changes were statistically significant in isolated CN3 palsy with *p* = 0.044 and *p* = 0.049, respectively.

The eight subjects with isolated CN4 palsy reported an initial preoperative AOD of 3.24° ± 2.22/3.62° ± 2.7 (near/distance) which changed to 2.86° ± 2.49/2° ± 2.02 (near/distance) and to 1.71° ± 1.23/1.43° ± 0.4 (near/distance) at the subsequent follow-up (near: *p* = 0.155, Cohen’s d = 0.95 (95% CI: −0.31, 2.12), distance: *p* = 0.626, Cohen’s d = 0.47 (95% CI: −1.1, 1.89)).

30 patients had isolated CN6 palsy and showed statistically significant improvement after surgery from 18.5° ± 8.25 and 18.48° ± 7.19 for near and distance preoperatively to 4.73° ± 4.74/4.35° ± 4.29 (near/distance) at first follow-up and to 5.52° ± 5.12/5.97° ± 4.47 (near/distance) at long-term follow-up (both *p* < 0.001).

**Figure 1:**
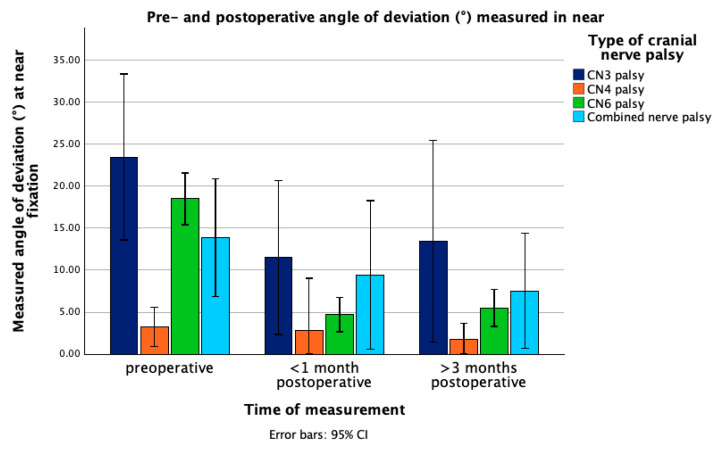
Mean angle of deviation (°) at near fixation in patients with different types of cranial nerve palsy, measured preoperatively, within one month postoperatively, and >3 months postoperatively. Error bars represent 95% confidence interval. Abbreviations: CN3 = third cranial nerve, CN4 = fourth cranial nerve, CN6 = sixth cranial nerve, CI = confidence interval.

**Figure 2:**
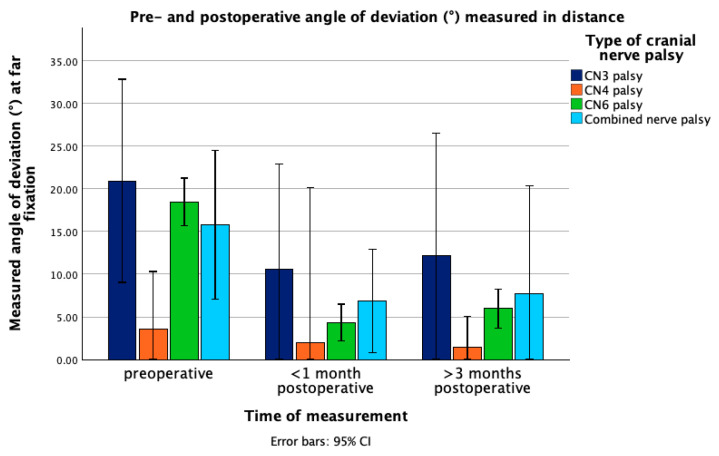
Mean angle of deviation (°) at far fixation in patients with different types of cranial nerve palsy, measured preoperatively, within one month postoperatively, and >3 months postoperatively. Error bars represent 95% confidence interval. Abbreviations: CN3 = third cranial nerve, CN4 = fourth cranial nerve, CN6 = sixth cranial nerve, CI = confidence interval.

AOD measured in near in patients with combined nerve palsy was 13.89° ± 7.57 preoperatively, 9.43° ± 8.44 at first follow-up and 7.54° ± 5.53 at second follow-up (*p* = 0.175, Cohen’s d = 0.74 (95% CI: −0.30, 1.71)). AOD measured in distance showed improvement from 15.77° ± 7.03 to 6.86° ± 5.77 at the first follow-up and to 7.71° ± 7.94 at the second follow-up. The change between preoperative AOD and at first follow-up was statistically significant (*p* = 0.022) but not between the preoperative value and AOD at second follow-up (*p* = 0.166, Cohen’s d = 1.23 (95% CI: −0.41, 2.77)). Figure 1 and Figure 2 depict the pre-and postoperative changes in the angle of deviation measured in near and distance in the different types of nerve palsy.

### 3.3. Change in Vertical Deviation

Mean preoperative VD measured in near in patients with isolated CN3 palsy improved from 6.86 (IQR 10.43) to 2.29 (15.86) after surgery (*p* = 0.068, r = 0.82). VD measured in distance changed in subjects with isolated CN3 palsy from 3.43 (7) to 2.29 (5.29) postoperatively (*p* = 0.109, r = 0.80).

Patients with isolated CN4 palsy demonstrated a preoperative VD of 3.14 (8.57)/2.29 (2.29) (near/distance) that changed to 2.57 (4.57)/2.86 (6.71) (near: *p* = 0.686, r = 0.18), distance: *p* = 0.257, r = 0.57).

The change in the mean preoperative VD was not statistically significant in patients with isolated CN6 palsy. It showed minimal change from 2.29 (6.29)/1.43 (4.29) to 3.14 (4.93)/2.29 (3.43) after surgery for near and distance, with *p* = 0.612 (r = 0.18) and *p* = 0.345 (r = 0.39), respectively.

Patients with combined nerve palsy showed a postoperative development from 8 (6.57) preoperatively to 4.57 (2.86) in VD measured in near (*p* = 0.116, r = 0.64). The improvement from 6.86 (5.43) to 2.86 (4.29) for VD measured in distance was statistically significant with *p* = 0.043.

### 3.4. Change in Refractive Error

The refractive error was measured by the spherical equivalent and changed from 0.19 ± 1.99 preoperatively to 0.14 ± 2.03 after surgery in patients with isolated CN3 palsy (*p* = 0.443, Cohen’s d = 0.25 (95% CI: −0.38, 0.88)). Patients with isolated CN4 palsy demonstrated a refractive error of −0.97 ± 1.4 preoperatively, which improved to −0.89 ± 1.38 post-surgery (*p* = 0.674, Cohen’s d = −0.17 (95% CI: −0.91, 0.59)). The change from −0.75 ± 2.94 before surgery to −0.55 ± 2.51 after surgery in patients with isolated CN6 palsy was not statistically significant (*p* = 0.321, Cohen’s d = −0.18 (95% CI: −0.54, 0.18)). The refractive error of the seven patients with combined nerve palsy minimally changed from 0.27 ± 1.01 to 0.29 ± 1.1 (*p* = 0.818, Cohen’s d = −0.09 (95% CI: −0.83, 0.66)).

### 3.5. Change in Visual Acuity

Preoperative visual acuity showed improvement from 0.09 (0.43) logMAR preoperatively to 0.03 (0.48) logMAR after surgery in patients with isolated CN3 palsy (*p* = 0.180, r = 0.45).

Preoperative VA in patients with isolated CN4 palsy remained very good, with pre- and postoperative values of 0.0 (0.07) and 0.0 (0.1), respectively (*p* = 0.581, r = 0.21).

Patients with isolated CN6 nerve palsy initially exhibited a VA of 0.08 (0.15), which changed to 0.1 (0.2) postoperatively (*p* = 0.809, r = 0.05).

VA in patients with combined nerve palsy remained stable from 0.05 (0.18) to 0.05 (0.06) after surgery (*p* = 0.197, r = 0.53).

### 3.6. Additional Operations

Two patients with isolated CN3 palsy underwent surgery on the MR and LR after a mean interval of 11.37 ± 2.87 months. One patient with isolated CN4 palsy received a revision on the inferior oblique muscle after 3.97 months. Five patients with isolated CN6 palsy also underwent reoperation after a mean of 32.73 ± 58.47 months. Two out of the five patients had MR recession and LR plication, one had LR plication, one had surgery on the superior rectus muscle and one had a Hummelsheim procedure.

### 3.7. Multivariable Logistic Regression

Multivariable logistic regression revealed no significant association between individual predictors and postoperative surgical outcome with *p*-values ranging from 0.061 (CN3 palsy) to 0.928 (vascular disease). No interaction terms were included in the final regression model. The results are listed in Table 5 with the *p*-value, Odds ratio (OR) and the 95% upper (UL) and 95% lower limit (LL).

## 4. Discussion

This study aimed to analyze the postoperative outcome of strabismus surgery in patients with nerve palsy to shed more light on the influencing factors and indications for surgical intervention.

In our study of all isolated types of nerve palsy, CN6 palsy was the most common, with 52.63% followed by isolated CN3 palsy (21.05%) and lastly isolated CN4 palsy (14.04%). Other studies have reported a similar distribution, describing CN6 palsy as the most prevalent and CN4 palsy as the least often type [2,25]. The most common etiologies of isolated CN3 palsy were vascular diseases and brain neoplasm, with 33.33% each. Although other studies on isolated CN3 palsy reported a higher rate of 52.7% of vascular causes and a lower rate of brain lesions (20.4%), the overall predominance of vascular and neoplastic etiologies is comparable to our findings [26,27].

25% of isolated CN4 palsies were congenital, while 50% of cases were caused by trauma, which is reaffirmed by other studies that list trauma as the most common etiology for isolated CN4 palsy [28]. In isolated CN6 palsy in our study, brain neoplasm was the most common determined cause (40%), followed by vascular diseases (20%) and traumatic incidents (16.67%). The results are broadly comparable to those of a Korean study on the etiologies of isolated CN3, CN4, and CN6 palsy, which concluded that brain neoplasm was the most common cause of the isolated CN6 palsies with a percentage of 25.4% [29]. In cases of combined nerve palsy, vascular disease accounted for 57.14%. This predominance may be explained by the fact that the complete nerve palsy was most often CN6 palsy, which is frequently associated with vascular causes [30].

The most common procedure in our study was recession and plication of the horizontal rectus muscle with or without additional oblique muscle recession (59.65%). Several other studies reported success in patients with isolated CN3 or CN6 palsy with horizontal recession and resection, which is the most straightforward and predictable technique [9,31]. It has to be noted that we used plication instead of resection as it is a less traumatic alternative to the latter, associated with less disruption and adverse postoperative reactions while obtaining similar good results [32,33]. The Hummelsheim procedure was used in large-angle AOD as the three patients with isolated CN6 palsy that underwent the technique had a larger mean preoperative AOD of 23.57° ± 7.25 compared to the mean of all isolated CN6 palsy cases (18.78° ± 7.67) [34]. A typical clinical presentation of isolated CN4 palsy includes head tilt to the opposite side, ipsilateral IO overaction and excyclodeviation [17]. Weakening of the overactive IO muscle through recession is the most commonly performed surgical technique for isolated CN4 palsy and was also done in most of the patients in this study (62.5%) [35,36]. The rest (37.5%) of the patients showed no elevation in adduction and underwent SO muscle plication, which is also said to yield more favorable results regarding residual head tilt [35].

Change in AOD after surgery was significant in patients with isolated CN3, isolated CN6, and combined nerve palsy when measured in distance, but not in patients with isolated CN4 palsy, and in patients with combined nerve palsy when measured near. VD was only statistically significant in patients with combined nerve palsy when measured in distance (*p* = 0.043). These findings can be explained by the clinical manifestation of the respective type of nerve palsy. CN3 palsy affects four extraocular muscles and primarily results in horizontal deviation, even though hypotropia is also present [7]. Merino et al. [9] obtained similar preoperative values with 40.24 PD (23°) and 44.29 PD (25.3°) for horizontal AOD measured in near and distance and 14.33 PD (8.19°) for VD in patients with isolated CN3 palsy. CN6 palsy affects the lateral rectus muscle, resulting in horizontal esodeviation and diplopia [37]. The surgical intervention primarily addresses the horizontal deviation and leads to a significantly improved AOD after surgery, while VD is not greatly changed. Isolated CN4 palsy, which affects the superior oblique muscle, primarily results in vertical deviation and less in horizontal deviation. In contrast to the findings reported by Yumuşak et al. [38] who analyzed the outcome of 27 patients, vertical deviation did not significantly change after surgery in CN4 palsy in this study, which may be due to the small sample size of 8 patients. We therefore propose further studies with a bigger patient group to analyze postoperative outcomes in CN4 palsy in greater detail.

No statistical significance could be detected between the pre- and postoperative refractive error in all patient groups and between pre- and postoperative BCVA. BCVA in CN4 palsy remained unchanged pre-and postoperatively (*p* = 0.581). Head tilt, which was present in 50% of cases, is a common compensatory mechanism in CN4 palsy. Patients with isolated CN4 palsy adopt the abnormal head posture to compensate for the hypertropia and to significantly decrease vertical misalignment [39,40]. Similarly, patients with combined nerve palsy maintained good BCVA with minimal postoperative change (*p* = 0.197).

We could not identify any preoperative parameter as highly predictive for surgical outcome, confirming the results of previous studies conducted on patients with nerve palsy that reported similar results [9,41]. In contrast, other studies on isolated CN6 palsies revealed a smaller preoperative AOD to be significantly associated with good surgical outcomes [31,42]. In our study, preoperative AOD was also not statistically significant (*p* = 0.149, OR 0.912, 95% CI: 0.804–1.034). 27 patients with isolated CN6 palsy obtained surgical success, more than half of them (60%) had a preoperative AOD of more than 15°. We hypothesize that this may be due to the complex neurological component of the nerve palsies and potential underlying pathologies, where strabismus is a secondary manifestation. Consequently, the preoperative AOD does not solely determine long-term success.

In our multivariable regression model, CN3 palsy showed a trend toward reduced odds of surgical success compared to CN6 palsy (*p* = 0.061, OR 0.143, 95% CI: 0.019–1.097). This suggests that management of CN3 palsy may be particularly challenging due to the involvement of multiple extraocular muscles. However, this association did not reach statistical significance, likely due to the small sample size of 12 patients. CN4 palsy (*p* = 0.201, OR 0.093, 95% CI: 0.002–3.535) and combined palsy (*p* = 0.123, OR 0.138, 95% CI: 0.011–1.708) also demonstrated reduced odds of surgical success, but with wide confidence intervals. Regarding etiology, no statistically significant difference could be found when comparing vascular disease, trauma and other causes with neoplasm as the reference category.

Our findings are generally comparable to previously reported outcomes of strabismus surgery due to cranial nerve palsy, even though follow-up periods and the definition of success varied between the studies. For CN3 palsy, we obtained a success rate of 58.33%, slightly lower than the 64.5% reported by Merino et al. [9] in their analysis of 31 cases. The discrepancy could be explained by the high number of complete CN3 palsies in our study (75% vs. 22.6%), as surgical management of complete palsy may be more challenging due to the lack of residual muscle activity. Success rates were higher in CN4 palsy with 87.5%, similar to the findings of Bagheri et al. [43], who reported that 84% of 73 patients undergoing surgery due to CN4 palsy had acceptable improvements. The definition of success was broader in the other study, as it included improvement of symptoms and abnormal head position, as well as a deviation of <5 PD, while we primarily focused on the postoperative alignment. In CN6 palsy, we obtained a higher success rate (90%) than the 67.3% reported in the previous study [41]. This is likely due to the different methodological approaches, as the other study focuses on outcomes at >12 months, whereas we had a shorter follow-up period. We furthermore defined success as <10° AOD, which is a less strict margin than the <10 PD criterion applied in the other study.

To the best of our knowledge, this is the first study to include an analysis of strabismus surgery due to combined nerve palsy. While our results are promising with 71.43%, the small sample size limits generalizability.

Inevitably, our study has several limitations. Due to the retrospective design, some patient groups, especially those with isolated CN4 palsy and combined nerve palsy, were small, which could limit statistical power and did not allow for a conclusive statistical analysis. The presented results should be interpreted cautiously.

Additionally, the mean follow-up period was relatively short and variable with 10.8 ± 15.38 months. This could influence outcomes, given that postoperative drift can occur. Nevertheless, most postoperative drifts are known to occur within the first year, which can be sufficiently captured with the current follow-up period [44].

Future studies would benefit from a standardized, long-term follow-up and a larger, randomized patient collective from multiple centers to reduce selection bias and improve generalizability. Including patient-reported outcomes, such as diplopia scores or quality-of-life measures, would further strengthen the results. Adding additional sensory functional endpoints, such as binocular single vision, would provide more insight into the postoperative results as helpful outcome measures in future studies.

Despite these limitations, this study provides a detailed analysis of the strabismus management due to cranial nerve palsy and adds meaningful insight to the current literature. Due to the rarity of the condition, existing studies on the different cranial nerve palsies remain limited. By reporting the surgical dosages and outcome measures in detail, our study offers a comprehensive overview of the surgical treatment of CN3, CN4 and CN6 palsies.

## 5. Conclusions

The analysis highlights several key points. Surgery should be tailored to the pattern of deviation. Most common techniques include horizontal two-muscle surgery for horizontal deviation and inferior oblique muscle recession for vertical deviation. Overall, surgery yields satisfactory results in 80.7% of cases. Surgical success varied with the different nerve palsies. CN6 palsy had the most favorable outcomes, whereas CN3 palsy proved to be the most challenging type. Significant postoperative improvements were obtained in horizontal deviations for CN3 and CN6 palsy, further in combined nerve palsy when measured in distance. Change in VD in combined nerve palsy was statistically significant when measured in distance. The lack of change in refractive error and visual acuity indicates that the benefit of the surgery is the motoric improvement rather than sensory changes. Clinically, surgeons can expect satisfactory outcomes regardless of age, gender, preoperative AOD, nerve subtype or etiology, but should consult the patient about the variability in success depending on the type of cranial nerve palsy.

## Figures and Tables

**Table 1:** Etiologies according to the type of nerve palsy.

Etiologies		CN3 Palsy (*n* = 12)	CN4 Palsy (*n* = 8)	CN6 Palsy (*n* = 30)	Combined Palsy (*n* = 7)
Congenital		0	2	0	0
Acquired	Inflammation	0	0	1	0
	Trauma	3	4	5	1
	Brain neoplasm	4	1	12	2
	Vascular disease	4	0	6	4
Unknown		1	1	6	0

Abbreviations: CN3 = third cranial nerve, CN4 = fourth cranial nerve, CN6 = sixth cranial nerve.

**Table 2:** Surgical procedures according to the type of nerve palsy.

Surgical Procedure	CN3 Palsy (*n* = 12)	CN4 Palsy (*n* = 8)	CN6 Palsy (*n* = 30)	Combined Palsy (*n* = 7)
MR recession & LR plication			20	1
LR recession & MR plication	9			1
Horizontal 2-muscle surgery with oblique muscle procedure	2		1	
MR recession			1	
LR plication			5	2
LR recession with oblique muscle recession	1			
LR plication with oblique muscle plication				2
IO recession		5		
SO plication		3		1
Hummelsheim procedure			3	

Abbreviations: CN3 = third cranial nerve, CN4 = fourth cranial nerve, CN6 = sixth cranial nerve, IO = inferior oblique muscle, LR = lateral rectus muscle, MR = medial rectus muscle, SO = superior oblique muscle.

**Table 3:** Surgical dosage ranges according to the type of nerve palsy.

Type of Strabismus	Preoperative AOD (°)	MR Recession/Plication * (mm)	LR Recession/Plication * (mm)
CN3 palsy	10	5.5	8
	15	5.5	9
	20	6	8
	50	6	12
			
CN4 palsy	2–14 VD	IO Recession: 11–13 mm with additional anteriorization (1–2 mm)	
			
CN6 palsy	5	4.5	9.5
	15	3	7
	20	4	8.5
	30	5	10

* recession = weakening procedure, plication = strengthening procedure; the procedure applied to the respective muscle depends on the type of palsy. Abbreviations: AOD = angle of deviation, CN3 = third cranial nerve, CN4 = fourth cranial nerve, CN6 = sixth cranial nerve, IO = inferior oblique muscle, LR = lateral rectus muscle, MR = medial rectus muscle, VD = vertical deviation.

**Table 4:** Demographic and clinical characteristics.

	CN3 Palsy (*n* = 12)	CN4 Palsy (*n* = 8)	CN6 Palsy (*n* = 30)	Combined Palsy (*n* = 7)
**Demographic factors**				
Age at time of surgery (years)	39.36 ± 27.65	32.21 ± 21.49	43.27 ± 22.38	46.49 ± 21.62
Gender				
Male	7 (58.33%)	5 (62.5%)	16 (53.33%)	2 (28.57%)
Female	5 (41.67%)	3 (37.5%)	14 (46.67%)	5 (71.43%)
**Clinical characteristics**				
Type of palsy				
Partial	3 (25%)	0 (0%)	1 (3.33%)	1 (14.29%)
Complete	9 (75%)	8 (100%)	29 (96.67%)	3 (42.86%)
Both *				3 (42.86%)
Preoperative AOD (°)				
Near	23.48 ± 14.7	3.24 ± 2.22	18.5 ± 8.25	13.89 ± 7.57
Distance	20.93 ± 14.24	3.62 ± 2.7	18.48 ± 7.19	15.77 ± 7.03
Postoperative AOD at first follow-up (°)				
Near	11.5 ± 12.8	2.86 ± 2.49	4.73 ± 4.74	9.43 ± 8.44
Distance	10.57 ± 14.72	2 ± 2.02	4.35 ± 4.29	6.86 ± 5.77
Postoperative AOD at second follow-up (°)				
Near	13.44 ± 15.61	1.71 ± 1.23	5.52 ± 5.12	7.54 ± 5.53
Distance	12.14 ± 17.15	1.43 ± 0.4	5.97 ± 4.47	7.71 ± 7.94
Preoperative refractive error (D)	0.19 ± 1.99	−0.97 ± 1.4	−0.75 ± 2.94	0.27 ± 1.01
Preoperative VA (logMAR (IQR))	0.09 (0.43)	0.0 (0.07)	0.08 (0.15)	0.05 (0.18)
Preoperative Ptosis	9 (75%)	-	-	1 (14.29%)
Preoperative Diplopia	8 (66.67%)	6 (75.00%)	12 (40%)	4 (57.14%)
Preoperative head tilt	2 (16.66%)	4 (50%)	4 (13.33%)	-
Surgical success	7 (58.33%)	7 (87.5%)	27 (90%)	5 (71.43%)
Failure	5 (41.67%)	1 (12.5%)	3 (10%)	2 (28.57%)

* combined cases with one complete nerve palsy and one partial nerve palsy. Abbreviations: AOD = angle of deviation, CN3 = third cranial nerve, CN4 = fourth cranial nerve, CN6 = sixth cranial nerve, D = diopters, IQR = interquartile range, logMAR = logarithm of the minimal angle of resolution, VA = visual acuity.

**Table 5:** Multivariable logistic regression.

Parameters		*p*-Value	OR	95% LL	95% UL
**Age**		0.314	1.019	0.983	1.056
**Gender**		0.902	0.894	0.150	5.322
**Preoperative AOD**		0.149	0.912	0.804	1.034
**Type of nerve palsy (with CN6 palsy as reference)**	CN3 palsy	0.061	0.143	0.019	1.097
	CN4 palsy	0.201	0.093	0.002	3.535
	Combined palsy	0.123	0.138	0.011	1.708
**Etiology (with neoplasm as reference)**	Vascular disease	0.928	0.899	0.089	9.101
	Trauma	0.880	1.191	0.123	11.509
	Other	0.628	0.545	0.047	6.345

Abbreviations: AOD = angle of deviation, CN3 = third cranial nerve, CN4 = fourth cranial nerve, CN6 = sixth cranial nerve, LL = lower limit, OR = Odds ratio, UL = upper limit.

## Data Availability

The data are not publicly available due to privacy concerns and ethical considerations. Access to the data can be requested from the corresponding author, subject to approval by the Ethics Committee of the Medical University of Vienna (protocol code EK Nr. 1260).

## Abbreviations

The following abbreviations are used in this manuscript:

AOD	Angle of deviation
BCVA	Best-corrected visual acuity
CN3	Third cranial nerve
CN4	Fourth cranial nerve
CN6	Sixth cranial nerve
D	Diopters
IO	Inferior oblique
IQR	Interquartile range
LL	Lower limit
logMAR	logarithm of the minimal angle of resolution
LR	Lateral rectus
MR	Medial rectus
OR	Odds ratio
PD	Prism diopters
SD	Standard deviation
SO	Super oblique
UL	Upper limit
VA	Visual acuity
VD	Vertical deviation
